# The average magnetic anisotropy of polystyrene in polymersomes self-assembled from poly(ethylene glycol)-*b*-polystyrene†

**DOI:** 10.1039/d3sm01333b

**Published:** 2023-12-05

**Authors:** Roger S. M. Rikken, Sandra Kleuskens, Loai K. E. A. Abdelmohsen, Hans Engelkamp, Roeland J. M. Nolte, Jan C. Maan, Jan C. M. van Hest, Daniela A. Wilson, Peter C. M. Christianen

**Affiliations:** a High Field Magnet Laboratory (HFML – EMFL), Radboud University, Toernooiveld 7 6525 ED Nijmegen The Netherlands hans.engelkamp@ru.nl; b Institute of Molecules and Materials, Radboud University, Heyendaalseweg 135 6525 AJ Nijmegen The Netherlands

## Abstract

Using the diamagnetic anisotropy of polymers for the characterization of polymers and polymer aggregates is a relatively new approach in the field of soft-matter and polymer research. So far, a good and thorough quantitative description of these diamagnetic properties has been lacking. Using a simple equation that links the magnetic properties of an average polymer repeating unit to those of the polymer vesicle of any shape, we measured, using magnetic birefringence, the average diamagnetic anisotropy of a polystyrene (PS) repeating unit, Δ*χ*^PS^, inside a poly(ethylene glycol)–polystyrene (PEG–PS) polymersome membrane as a function of the PS-length and as a function of the preparation method. All obtained values of Δ*χ*^PS^ have a negative sign which results in polymers tending to align perpendicular to an applied magnetic field. Combined, the same order of magnitude of Δ*χ*^PS^ (10^−12^ m^3^ mol^−1^) for all polymersome shapes proves that the individual polymers are organized similarly regardless of the PS length and polymersome shape. Furthermore, the value found is only a fraction (∼1%) of what it can maximally be due to the random coiling of the polymers. We, therefore, predict that further ordering of the polymers within the membrane could lead to similar responses at much lower magnetic fields, possibly obtainable with permanent magnets, which would be highly advantageous for practical applications.

## Abstract 2

The magnetic response of polymer vesicles can be studied using magnetic birefringence, but its response is hampered by a large degree of disorder in the polymer membrane. Here we show that a universal effective anisotropy per monomer remains, which can be used to study different polymersome shapes.

## Graphical abstract

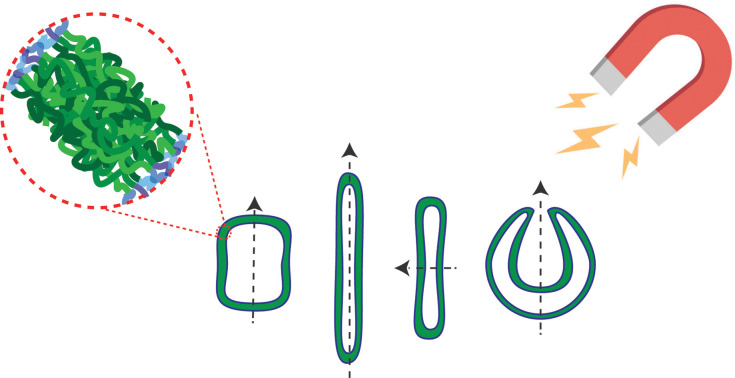

## Introduction

In the past decades, polymer vesicles, or polymersomes, have gained tremendous importance in the research fields of micro/nanoreactors, drug delivery and cell mimicking.^1^ Polymersomes are self-assembled structures composed of amphiphilic block-copolymers, which can easily be adapted to control the physical, chemical and biological properties of the vesicle.^1^ This allows for great tunability of polymersomes for possible medical applications. Controlling the shape of the polymersomes is a crucial step for applications *in vivo*.^2^ In order to fully understand the shape of the assemblies, more attention needs to be paid to the physical properties of the block copolymer. In this paper, we will focus on the diamagnetic properties of polymersomes.

Diamagnetic assemblies have great potential for applications in magnetic fields.^3^ When the diamagnetic susceptibility of an object is anisotropic, the object will have a preferential direction in a magnetic field, which leads to magnetic alignment.^4^ By aligning particles, some directional control over particle motion is achieved for autonomously moving particles.^5,6^ The magnetic response of particles originates from the chemical constituents. The chemical structure of a molecule gives rise to diamagnetic anisotropy. The diamagnetic anisotropy has been determined for a large number of chemical bonds and groups.^7–11^ For molecular assemblies, the total diamagnetic anisotropy depends on the orientation of the molecules in the assembly, quantified with the order parameter. This diamagnetic anisotropy can also be used to characterize polymer aggregates and polymers, even though the monomers are largely randomly oriented. As our group has shown before, we are able to characterize polyethylene glycol–polystyrene (PEG–PS) polymersomes based on their diamagnetic anisotropy by using partial magnetic alignment.^12,13^ Thus far, the random arrangement of the monomers in atactic polystyrene hampers a quantitative description of the diamagnetic properties of PEG–PS polymersomes. If the quantitative description of the properties were known, the polymersomes could be adapted such that a higher magnetic response is achieved.

Over the last decade, several techniques have been developed to determine the diamagnetic anisotropy of material samples. The preferred technique heavily depends on the properties of the materials and the size of the sample. Experimentally, the diamagnetic anisotropy of a variety of relatively large (macroscopic) crystals has been determined by oscillating them in a static magnetic field, using either fibers for the suspension or micro-gravity to keep the crystals in place.^14–17^ Diamagnetic anisotropies have also been detected during phase transitions by magnetically levitating a sample while heating or cooling it.^18^ Nuclear magnetic resonance (NMR) has been used to determine the diamagnetic anisotropies of single bonds or small chemical groups.^19,20^ For small molecular aggregates in solution, the diamagnetic anisotropy can be determined by measuring the degree of alignment as a function of the applied magnetic field, often by using optical techniques such as magnetic birefringence (MB)^13,21–24^ and polarized absorbance.^25^ MB has been used to determine the magnetic alignment of a wide variety of biological and organic structures.^26–30^

For molecular aggregates in solution, such as our polymersomes, the first two mentioned techniques are not always well suited. Molecular aggregates are usually too small to measure with the magnetic oscillation technique, while NMR usually focuses strongly on individual chemical bonds or groups. For our PEG–PS polymersomes, the diamagnetic anisotropy of the individual polymer depends on its local environment in its assembly. Therefore, a technique is preferred in which the magnetic anisotropy of the polymer vesicle is measured as a whole, which in turn can be related to that of a single polymer. To this end, MB is the most preferred technique.

In this research, the aim is to establish the relationship between the magnetic response of a rigid polymersome shape in high magnetic fields and its shape. Based on this relationship, the magnetic properties of the polymer and the alignment direction of the polymer and various polymer shapes can be determined. First, we discuss the theory of the diamagnetic susceptibility of polymersomes, after which we study self-assembled polymersome shapes, made out of different polymer lengths, using MB. Combining MB with electron microscopy, we determined the average magnetic anisotropy of the polystyrene monomer in the polymersome membrane. This value gave us insight into polymer packing in the polymersome membrane.

## Theory

Anisotropic objects, having anisotropic magnetic susceptibility, tend to align in a magnetic field. The degree of alignment can be described by the order parameter *S*1
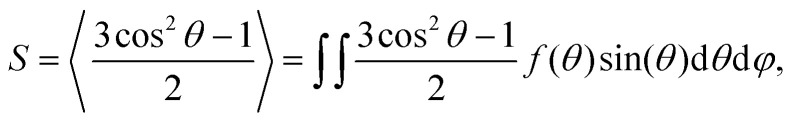
where *θ* is the polar angle between the object's symmetry axis and the field lines, taken to be in the *z*-direction, *ϕ* is the angle of the object's projection makes with the *xy*-plane and *f*(*θ*) is the Boltzmann distribution function defined as2
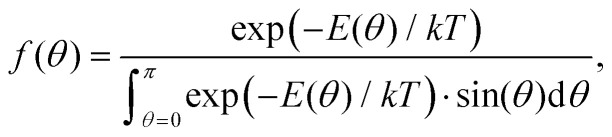
with3
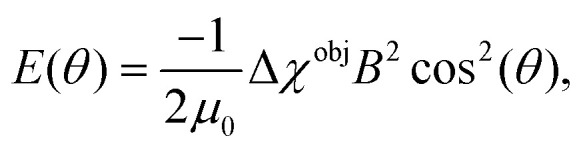
as the magnetic energy of an object as function of *θ, k* is the Boltzmann constant, *T* is the temperature, *μ*_0_ is the magnetic permeability of free space, *B* is the applied magnetic field strength and Δ*χ*^obj^ is the magnetic anisotropy of the object to be aligned.^31^ Magnetic alignment of anisotropic particles usually leads to a difference in refractive index relative to the magnetic field direction Δ*n* = *n*_‖_ − *n*_⊥_ which can be measured by magnetic birefringence. MB depends proportionally on the order parameter by4
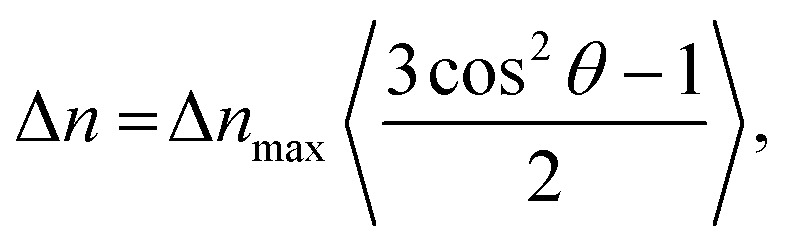
where Δ*n*_max_ is the maximal birefringence reachable at complete alignment. MB is therefore frequently used to measure the degree of alignment.^4,13,21,22,26–30^

**Fig. 1:**
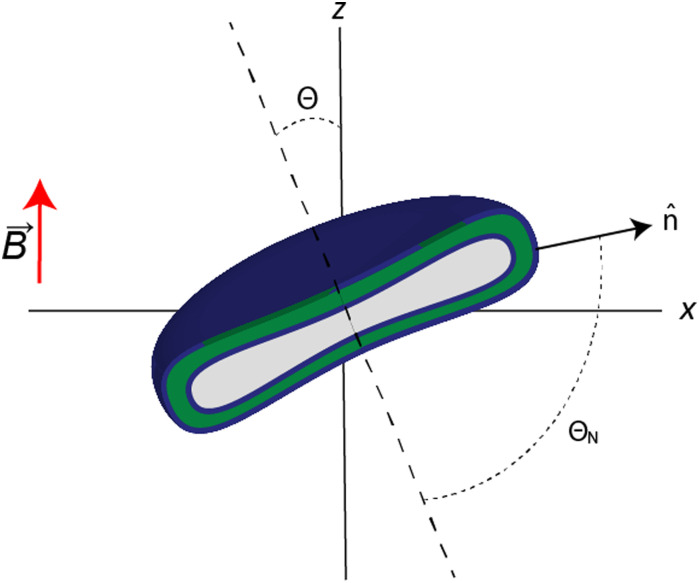
Schematic picture of a polymersome disc in a magnetic field indicating the difference between the angle *θ*, which is related to the alignment of the vesicle's symmetry axis (dashed line) in a magnetic field (red arrow), and the angle *θ*_N_, which is related to the direction of the membrane (normal vector n̂) with respect to the vesicle's symmetry axis.

The magnetic anisotropy of a polymer vesicle with cylindrical symmetry can be defined as Δ*χ*^ves^ = *χ*_‖_ − *χ*_⊥_, where *χ*_‖_ and *χ*_⊥_ are the tensor components parallel and perpendicular to the symmetry axis, respectively. The magnetic anisotropy of this vesicle is size and shape dependent, as we have already experimentally demonstrated previously.^13^ Therefore, it is highly advantageous to express the magnetic susceptibility of a vesicle in terms of its molecular constituents, which is identical for all polymersome shapes and sizes. We have derived (Supporting Information 1 and Fig. S1, ESI†) the relationship between the anisotropic part of the magnetic susceptibility of a vesicle, Δ*χ*^Ves^, and that of a single average polystyrene repeating unit, Δ*χ*^PS^, in the vesicle's membrane to be:5
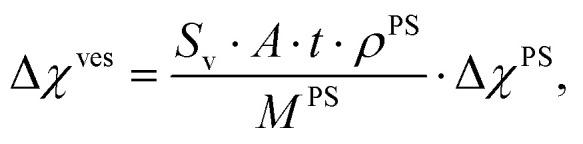
where *A* is the surface area of the polymersome, *t* is the thickness of the membrane, *ρ*^PS^ is the density of the polystyrene part of the membrane (1055 kg m^−3^),^32^*M*^PS^ is the molecular weight of a single polystyrene repeating unit (1.73 × 10^−25^ kg) and *S*_V_ is a shape-dependent order parameter,6
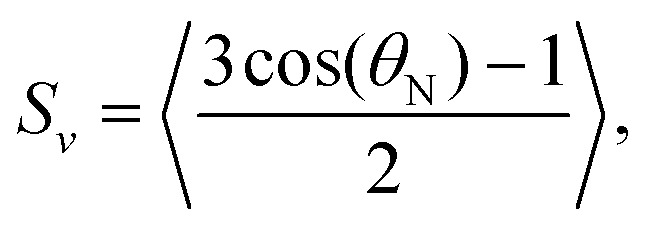
where *θ*_N_ is the angle between the normal vector of the membrane and the vesicle's symmetry axis (see Fig. 1). Substituting eqn (5) into eqn (3) gives7
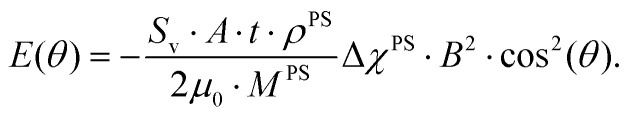
A value of Δ*χ*^PS^ can then be determined by fitting eqn (4); to measure the MB curve, the shape and size dependent parameters, *S*_V_, *A* and *t*, are provided, which can all be determined with electron microscopy.

## Results

**Fig. 2:**
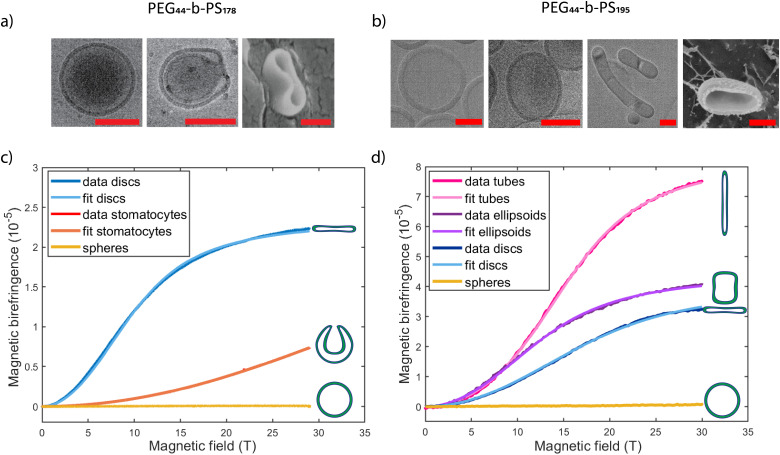
Examples of electron microscopy images of a (a) PEG44-*b*-PS178 sphere (left, cryo-TEM), stomatocyte (middle, cryo-TEM) and disc (right, cryo-SEM) and (b) PEG44-*b*-PS195 sphere (cryo-TEM), disc (cryo-SEM), ellipsoid (cryo-TEM), and tube (cryo-TEM). All images have a scale bar of 250 nm and show the cross section of the shape, allowing all of them to be parameterized as shown in ESI,† Fig. S4–S8. (c) Magnetic birefringence of PEG44-*b*-PS178 polymersome discs, stomatocytes and spheres. For discs and stomatocytes, the best fit of Δ*χ*^PS^ is included. The sign of Δ*χ*^PS^ is negative for both fits. The spheres do not show any MB signal as is to be expected for aggregates of spherical symmetry (identical to water). The MB of the discs is almost fully saturated at 29 T while the MB of the stomatocytes is not. (d) Magnetic birefringence of PEG44-*b*-PS195 polymersome tubes, ellipsoids, discs and spheres. For tubes, ellipsoids and discs, almost full alignment was observed at 30 T and the best fits of Δ*χ*^PS^ are included. The sign of Δ*χ*^PS^ is negative for all shapes. The error bars are of the order of the thickness of the line as well as the goodness of all fits is shown in ESI,† Table S1.

**Fig. 3:**
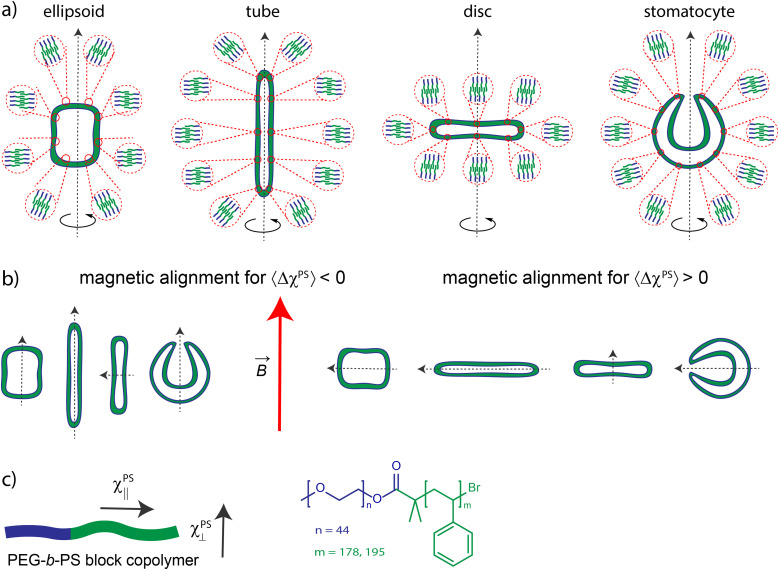
(a) Schematic images of an ellipsoid, a tube, a discs and a stomatocyte. The distributions of the individual polymers are indicated at several positions. The ellipsoid, tube and stomatocyte have most polymers perpendicular to the vesicles’ symmetry axis (dashed arrow), while the disc has most polymers parallel to its symmetry axis. Therefore, a disc and an ellipsoid, a tube or a stomatocyte will align with their symmetry axis in opposite directions. The two possibilities are shown in (b). (c) Legend also shows how the magnetic anisotropy of polystyrene (PS) is defined with respect to the polymer.

Here, rigid polymersome shapes were obtained by applying three different techniques to fabricate self-assembled polymersomes. For PEG44-*b*-PS178 polymersomes, stomatocytes were formed by dialysis,^13^ whereas discs were created by an out-of-equilibrium technique.^12^ The polymer addition technique was used to prepare PEG44-*b*-PS195 ellipsoids, tubes and discs by increasing the osmotic pressure applied by the added polymer, resulting in fast shape transformation (<4 min).^33^ TEM, Cryo-TEM and Cryo-SEM were used to image all polymersome samples. A representative image of every shape is shown in Fig. 2a and b (more images can be found in Fig. S2–S8, ESI†). Cryo-TEM images were used to determine the thickness, *t*, of the membrane for all shapes, which was found to be 26 ± 3 nm, independent of the vesicle shape and the polymer length. To obtain quantitative information about the shape and size of all polymersome samples, we fitted five different vesicles for each shape, using the parameterization postulated previously^12^ (see the Supporting Information 1, ESI†). All individual fittings and fitting parameters are given in Fig. S4–S8 and Tables S2–S6 (ESI†). It was found that the sign of *S*_V_ was negative for the stomatocytes and tubes, and positive for the discs and ellipsoids. This is because in stomatocytes, tubes and ellipsoids a majority of the polymers are pointing perpendicular to the vesicle's symmetry axis while in the discs most of the polymers are oriented parallel. This is shown schematically in Fig. 3a. As a result, the alignment of polymersomes in a magnetic field occurs in different directions: some shapes align with their symmetry axis parallel to the magnetic field while others align with their symmetry axis perpendicular to the magnetic field. The sign of Δ*χ*^PS^ determines the orientation of the symmetry axis relative to the direction of the magnetic field, as is shown in Fig. 3b. With *S*_V_·*A*, and *t* is determined for both the discs and the stomatocytes, Δ*χ*^PS^, and its sign can be determined by fitting the MB curves.

The magnetic birefringence was measured for all rigid polymersome samples. In Fig. 2c, the sample consisting of spheres shows no measurable MB up to 29 T compared to the background (water). This is to be expected since spheres are completely isotropic with no preferred direction of alignment. The stomatocytes have a small anisotropy, which is confirmed by the small MB, even up to 29 T. The largest signal was obtained by the most anisotropic shape (discs). Not only do the discs give the highest MB, the MB is also observed to saturate, indicating that the magnetic alignment is almost complete at 29 T. For the PEG44-*b*-PS195 samples, the magnetic birefringence is shown in Fig. 2d. Again, no significant change was observed for spherical-shaped polymersomes. The highest signal is obtained for tubes, followed by ellipsoids and discs, whereas most alignment was found for discs, followed by ellipsoids and tubes.

In order to obtain Δ*χ*^PS^, the MB curves of all polymersome shapes were fitted using eqn (1) with *f*(θ), S_V_ and *E*(θ) given by eqn (2), (6) and (7) respectively. This allowed us to directly fit Δ*χ*^PS^, which is the only fitting parameter, to the MB data. The other parameters have been obtained *via* electron microscopy. The best fits are shown in Fig. 2c and d and the corresponding values of Δ*χ*^PS^ obtained are listed in Table 1. For PEG_44_-*b*-PS_178_ polymersomes, the values of Δ*χ*^PS^ are equal for both stomatocytes and discs. This is expected as both particles were made from identical polymers and the shape transformation process allowed sufficient time for rearrangement. For PEG_44_-*b*-PS_195_ polymersomes, the absolute Δ*χ*^PS^ value decreased with the amount osmotic pressure applied.^33^ The biggest value was found for the ellipsoids and the smallest value was found for the discs. Due to the fast shape transformation, the polymers in the membrane might have less time to rearrange, which could result in a different packing of polystyrene in the membrane. Comparing all the five values, Δ*χ*^PS^ is negative, has the same order of magnitude and seems to be independent on the polymer length. Taking the average of all these five values, Δ*χ*^PS^ is −2.9 × 10^−12^ ± 0.4 m^3^ mol^−1^.

**Table 1:** Results of the fittings of the birefringence curves for PEG_44_-*b*-PS_178_ and PEG_44_-*b*-PS_195_. The small value difference for PEG_44_-*b*-PS_195_ might be attributed to a different packing of polystyrene monomers in the membrane caused by the fast shape transformation

	Fitted Δ*χ*^PS^ for PEG44-*b*-PS178 (10^−12^ m^3^ mol^−1^)	Fitted Δ*χ*^PS^ for PEG44-*b*-PS195 (10^−12^ m^3^ mol^−1^)
Ellipsoids	n/a	−5.2 ± 1.2
Tubes	n/a	−2.9 ± 0.9
Discs	−2.7 ± 0.4	−1.4 ± 0.2
Stomatocytes	−2.5 ± 0.3	n/a

## Discussion

**Fig. 4:**
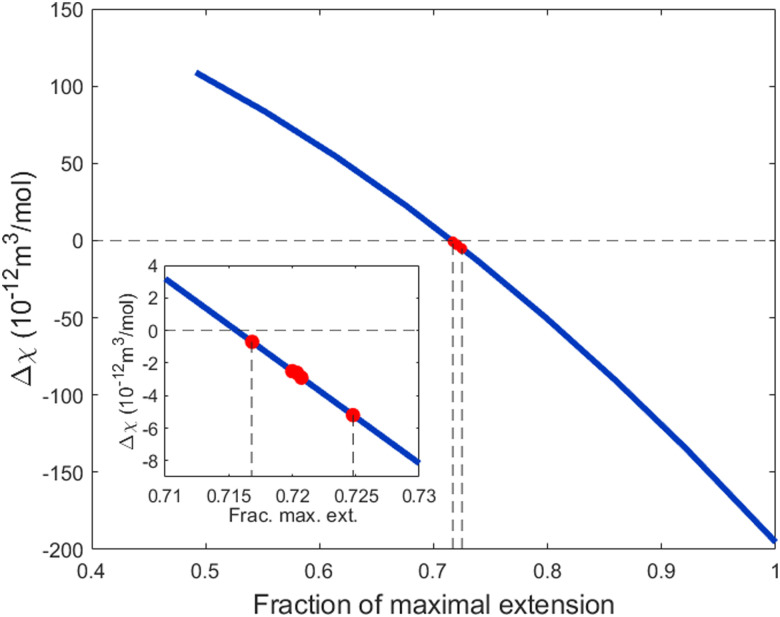
Δ*χ*^PS^ as a function of the extension of the polystyrene in the membrane. The experimentally determined values of Δ*χ*^PS^ are indicated with red dots. The inset is a magnification of the region around the red dots. The experimentally determined values are only a fraction of what can be obtained when fully stretching the polystyrene chains within the polymersome membrane.

The magnetic anisotropy of PS also provides insight into the polymersome membrane organization. In the polymersome membrane, most PS monomers are expected to be randomly orientated. Therefore, only a few PS monomers contribute to the effective Δ*χ*^PS^. These few monomers are expected to be at the edge of the membrane, where the polymer is forced to be orientated perpendicular to the membrane and the bending stiffness is the largest. Recent research showed that the bending stiffness (persistence length) of PS is 1 monomer,^34^ which implies that the rest of the PS monomers are expected to be coiled up. To determine the degree of coiling, Δ*χ*^PS^ is calculated as a function of the fraction of the maximal extension of PS in the polymersome membrane, assuming that phenyls are free to rotate around the bonds connecting them to the PS backbone (for more details on this calculation see the Supporting Information 2 and Fig. S9, ESI†). When the polymer is fully extended parallel to the membrane, the expected value for Δ*χ*^PS^ is 110 × 10^−12^ m^3^ mol^−1^, whereas the maximal perpendicular extension gives −195 × 10^−12^ m^3^ mol^−1^ (Fig. 4). The experimentally determined values of Δ*χ*^PS^ correspond to an average extension of 72% as can be seen in Fig. 4. This is very close to 71.5% extension, where we find completely random orientation of the polymer and thus no diamagnetic susceptibility. For the polymer lengths used here, this 0.5% extraction difference of the PS backbone corresponds to 1 PS monomer not being randomly coiled up, which is in agreement with the persistence length of PS. Most of the polymersome membrane thus exist out of random coiled polymers where the one PS monomer at the surface is ordered and gives rise to the diamagnetic susceptibility.

Fig. 4 also shows the potential of tuning the ordering of the polymers in the polymersome membrane. When the PS chain is contracted, the sign of Δ*χ*^PS^ is flipped, which would give control over the direction of alignment (parallel or perpendicular to the applied magnetic field). The chain extension could result in a maximal increase in the magnetic anisotropy by a factor 75. By creating polymersomes consisting of strongly extended polymers, one would greatly lower the required magnetic field to align them. For instance, when the polymers are fully extended, fields of less than 1 T would already be sufficient to fully align a disc-shaped polymersome. This would make applications more realistic since these kinds of fields can easily be generated with permanent magnets. Increasing the ordering of a diamagnetic material to reduce the required magnetic field strength for magnetic manipulation has already been proven to work before by the use of highly oriented pyrolytic graphite (HOPG), which can even be levitated using permanent magnets.^35–37^ Likewise, an increased ordering of the polymers within the polymersome membrane would lead to magnetic alignment at fields which are also obtainable with permanent magnets.

## Experimental

### Materials and instrumentation

Tetrahydrofuran and 1,4-dioxane were purchased from Sigma-Aldrich. The dialysis membranes were Spectra/Por Dialysis Membrane 4 (MWCO 12–14 000) from Spectrum Laboratories, Inc.

Magnetic birefringence measurements were performed using a standard polarization modulation technique.^4^ A 1.5 mW intensity stabilized HeNe laser (632.8 nm) was used from Research Electro-Optics Inc. to probe dispersion in a 5 mm thick quartz cuvette. The magnetic field was applied using a 33 T Bitter magnet at the High Field Magnet Laboratory.

Cryo-SEM was performed using a JEOL 6330 cryo-field emission scanning electron microscope at an acceleration voltage of 3 kV in cryo-mode. TEM was performed using a JEOL 1010 transmission electron microscope at an accelerating voltage of 60 kV, for which 4 μL of the sample was air dried on 200 Mesh carbon coated copper grids. Cryo-TEM was performed using a JEOL 2100 cryo transmission electron microscope. DLS was performed with a Malvern Zetasizer Nano S instrument and its data were analyzed with the corresponding software from Malvern Instruments.

### Polymersome sample preparation *via* dialysis

Spherical polymersomes were self-assembled as follows. First, 10 mg of PEG_44_-*b*-PS_178_ powder was dissolved in a mixture of 600 μL of tetrahydrofuran (THF) and 400 μL of 1,4-dioxane and the solution was stirred for 30 minutes. Next, 3 mL of water was added at a rate of 1 mL h^−1^ (while stirring at 750 rpm) at which point polymersomes were formed. Polymersome formation was observed by the transition from a clear colourless solution to a cloudy suspension. Afterwards, the organic solvents were removed by dialysis against pure water in a 12–14 kDa cutoff membrane tubing over 48 hours. During this time, water was replenished 5 times at regular intervals. The resulting sample consisted of spherical polymersomes in pure water.

Stomatocytes were obtained by applying the solvent addition technique on rigid spherical polymersomes as was described earlier.^38^ Discs were obtained by the out-of-equilibrium technique.^12^ The obtained stomatocytes and discs were rigidified by injecting them in an excess of water to quench the polystyrene membrane. The concentration was increased afterwards by performing spin filtration at 3000 rpm to 1 mg mL^−1^. The final concentration was determined by freeze-drying a fraction of the sample and weighing the amount of polymer in it.

### Polymersome sample preparation *via* polymer addition

PEG_44_-*b*-PS_195_ (10 mg) was dissolved in 1 mL of a 4 : 1 solvent mixture of tetrahydrofuran (THF) : 1,4-dioxane (dioxane) in a 15 mL capped vial with a magnetic stirrer and stirred for 30 minutes. A syringe pump equipped with a syringe with a needle was calibrated to deliver water at a speed of 1 mL h^−1^. The needle from the syringe was inserted into the vial and closed with a rubber septum. 1 mL of water was pumped into the organic solution accompanied by vigorous stirring (900 rpm). After self-assembly of the polymersomes was completed, polymersome suspensions of 150 μL were transferred into 1.5 mL Eppendorf tubes. 2 μL, 10 μL (10 mg mL^−1^) or 10 μL (100 mg mL^−1^) of PEG was added, shaked (1200 rpm) and transferred to the different Eppendorf tubes, and the spherical polymersomes were transformed into tubes, discs and stomatocytes, respectively. After 4 minutes, the samples were quenched in an excess of MilliQ and washed three times by centrifuging the suspension and refreshing the MilliQ water to obtain a sample with a final concentration of 1 mg mL^−1^.

## Conclusions

We have demonstrated that it is possible to determine the magnetic anisotropy of a single polystyrene monomer by measuring the MB of differently shaped polymersomes which are self-assembled from PEG_44_-*b*-PS_178_ and PEG_44_-*b*-PS_195_ block copolymers. The necessary information details for the MB fittings are the shape factor, surface area and membrane thickness, which can all be determined by cryo-EM and the parameterization of the obtained cross-sections. For vesicles self-assembled from PEG-*b*-PS polymers, we determined that the magnetic anisotropy of a single polystyrene monomer is far from the maximum that can be obtained by fully stretching the polymers within the membrane. Comparisons with the theoretical calculation also showed that the sign of Δ*χ*^PS^ can be flipped by even a small contraction of the polymers. Further research on how to control the extension of the block copolymers in a membrane could therefore lead to new methods that allow one to control the strength and/or direction of the magnetic response. This would be highly beneficial for applications since it could lead to a significant reduction of the required field strength.

## Conflicts of interest

There are no conflicts to declare.

## Supplementary Material

SM-020-D3SM01333B-s001
