# Supplementary material for: The average magnetic anisotropy of polystyrene in polymersomes self-assembled from poly(ethylene glycol)-b-polystyrene
Source: Soft Matter. 2023 Dec 5;20(4):730–7. doi: 10.1039/d3sm01333b (PMC10806999; doi:10.1039/d3sm01333b)
Supplement: SM-020-D3SM01333B-s001 [file SM-020-D3SM01333B-s001.pdf]

Electronic Supplementary Material (ESI) for Soft Matter.
This journal is © The Royal Society of Chemistry 2023

The average magnetic anisotropy of polystyrene in polymersomes self-assembled from poly(ethylene glycol)-*b*-polystyrene

Roger S.M. Rikken[‡a,b] Sandra Kleuskens, [‡a,b] Loai K.E.A. Abdelmohsen,[b] Hans Engelkamp,*[a,b] Roeland J.M. Nolte,[b] Jan C. Maan,[a,b] Jan C.M. van Hest,[b] Daniela A. Wilson[b] and Peter C.M. Christianen[a,b]

[a] High Field Magnet Laboratory (HFML – EMFL), Radboud University, Toernooiveld 7, 6525 ED Nijmegen, The Netherlands

[b] Institute of Molecules and Materials, Radboud University, Heyendaalseweg 135, 6525 AJ Nijmegen, The Netherlands

Supporting Information

Supporting Information 1: Derivation of surface parameter 2

Supporting Figure 1: Schematic representation of a disc. The magnetic anisotropy of the individual polymers and that of the vesicle as a whole are indicated. The angle θ_N *is indicated.* 3

Supporting Figure 2: TEM image of the discs. The whole sample consists of discs although there is some spread in the size of the discs. Scale bar is 1 µm. 5

Supporting Figure 3: TEM image of the stomatocytes. The whole sample consists of open stomatocytes although there is some small spread in the size and shape. Scale bar is 1 µm. 6

Supporting Figure 4: Cryo-SEM images showing disc cross sections. Every cross section has been fitted using the parameterization stated in ref. The fitting parameters and the corresponding surface parameter (SP) calculated are given in Supporting Table 1: Accuracy of the $\Delta\chi^{PS}$ *of the magnetic birefringence curves for* PEG_{44}*-b-*PS_{178} *and* PEG_{44}*-b-*PS_{195} .... ***Error! Bookmark not defined.***

Supporting Table 2: Fitting parameters and surface parameter (SP) for discs, obtained from the fittings as shown in figure 4. 7

Supporting Figure 5: Cryo-TEM images showing the cross sections of the stomatocytes. Every cross section has been fitted using the parameterization stated in ref. The fitting parameters and the corresponding surface parameter (SP) calculated are given in Supporting Table 2. 8

Supporting Table 3: Fitting parameters and surface parameter (SP) for the stomatocytes, obtained from the fittings as shown in figure 5. 8

Supporting Information 2: Calculation of $\Delta\chi^{PS}$ as function of polymer extension 9

Supporting Figure 6: Results of the calculation of the magnetic anisotropy of a single polystyrene repeating unit as function of the extension of the polystyrene polymer. 13

References 14

Supporting Information 1: Derivation of surface parameter

In order to calculate the magnetic properties of a polymersome vesicle, it is necessary to transform the magnetic susceptibilities of every single polymer in the membrane (both $\chi_{\parallel}^{P}$ and $\chi_{\perp}^{P}$) from the polymer axes to the axes in which the whole vesicle will be described (see Supporting Figure 1). This transformation gives:

$$\begin{aligned}
\chi_x^P &= \chi_{\parallel}^{P} \sin^2(\theta_N)\cos^2(\phi) + \chi_{\perp}^{P}\cos^2(\theta_N)\cos^2(\phi) + \chi_{\perp}^{P}\sin^2(\phi) \\
\chi_y^P &= \chi_{\parallel}^{P} \sin^2(\theta_N)\sin^2(\phi) + \chi_{\perp}^{P}\cos^2(\theta_N)\sin^2(\phi) + \chi_{\perp}^{P}\cos^2(\phi) \qquad \text{(S1)} \\
\chi_z^P &= \chi_{\parallel}^{P} \cos^2(\theta_N) + \chi_{\perp}^{P}\sin^2(\theta_N)
\end{aligned}$$

with θ_N defined as:

$$\theta_N = \cos^{-1}\left(\frac{n_z}{\hat{n}}\right) \qquad \text{(S2)}$$

and φ the angle describing the orientation around the vesicle's symmetry axis.

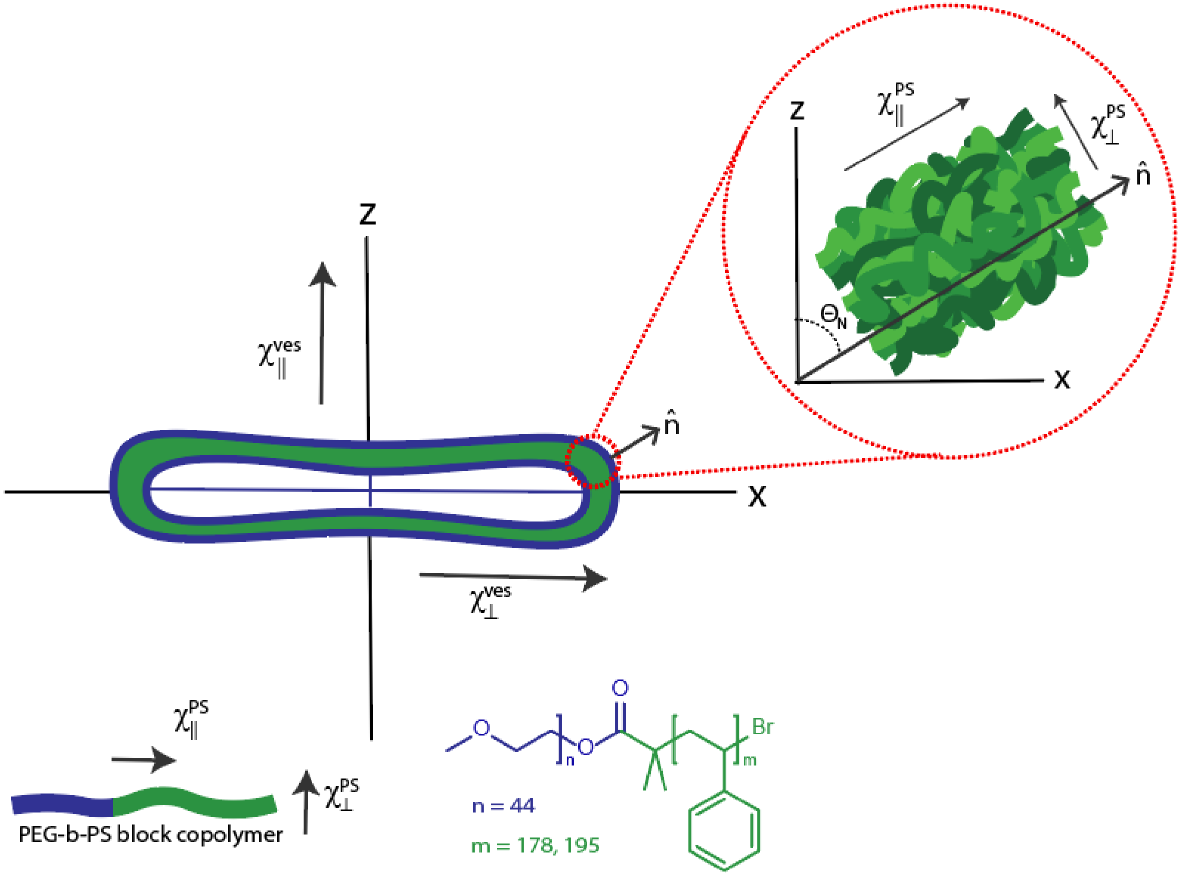

Supporting Figure 1: *Schematic representation of a polymersome disc and its internal organization. The magnetic anisotropy of the vesicle as a whole, the individual polymers are indicated. The angle θ_N is indicated. Only the PS chain is visualized as it dominates the magnetic anisotropy of the PEG-PS polymer.*

In a previous publication,[1] we formulated the following parametrization in which any cylinderically symmetric vesicle shape can be described:

$$x = \left(\sum_{n=1}^{l} a_n \sin(n \cdot v)\right) \cdot \cos(u) \quad \text{(S3)}$$

$$y = \left(\sum_{n=1}^{l} a_n \sin(n \cdot v)\right) \cdot \sin(u) \quad \text{(S4)}$$

$$z = \sum_{n=1}^{l} b_n \cos(n \cdot v) \quad \text{(S5)}$$

With v from 0 to π and u from 0 to 2π.

The magnetic anisotropy of the whole vesicle can be calculated by integrating the parametrization over the surface of the vescile. In terms of this parametrization this can be expressed as:

$$\Delta\chi^{ves} = \int_{u=0}^{2\pi}\int_{v=0}^{\pi}\left(\chi_z^P - \frac{\chi_x^P+\chi_y^P}{2}\right)\cdot\frac{N}{A}\cdot J(v)\cdot dv\cdot du \qquad \text{(S6)}$$

with N the number of polymers in the vescile, A the surface area of the vesicle and $J(v)$ the Jacobian of the parametrization used:[1]

$$J(v) = \begin{pmatrix} [\sum_{n=1}^{4} n\cdot b_n \sin(n\cdot v)\cdot\sum_{n=1}^{4} a_n \sin(n\cdot v)]^2 \\ +[\sum_{n=1}^{4} n\cdot a_n \cos(n\cdot v)\cdot\sum_{n=1}^{4} a_n \sin(n\cdot v)]^2 \end{pmatrix}^{0.5} \qquad \text{(S7)}$$

Equation (S6) can be shortened to:

$$\Delta\chi^{\mathrm{ves}} = 2\pi\int_{v=0}^{\pi}\left(\frac{3\cos^2(\theta_N)-1}{2}\right)\cdot\left(\chi_{\parallel}^{\mathrm{P}} - \chi_{\perp}^{\mathrm{P}}\right)\cdot\frac{N}{A}\cdot J(v)\cdot dv \qquad \text{(S8)}$$

since terms within the integral have no dependency in u due to their cylindrical symmetry. In general, the relation between $\Delta\chi^{\mathrm{ves}}$ and $\Delta\chi^P$ can be written as:

$$\Delta\chi^{\mathrm{ves}} = \langle\frac{3\cos^2(\theta_N)-1}{2}\rangle\cdot\Delta\chi^P\cdot N \qquad \text{(S9)}$$

Since the magnetic anisotropy of a PEG-PS block copolymer is dominated by the PS units, as was demonstrated before,[1] we can describe $\Delta\chi^{\mathrm{ves}}$ in terms of a the magnetic anisotropy of a single PS repeating unit, $\Delta\chi^{\mathrm{ves}}$, by:

$$\Delta\chi^P = m\cdot\Delta\chi^{\mathrm{PS}} \qquad \text{(S10)}$$

With m the number of PS units in a single PS polymer. The number of polymers in a vesicle N, can be written as function of surface area A, membrane thickness t, polystyrene density ρ^{PS} and weight of a PS repeating unit M^{PS} by:

$$N = \frac{A\cdot t\cdot\rho^{\mathrm{PS}}}{m\cdot M^{\mathrm{PS}}} \qquad \text{(S11)}$$

Combining equations (S9), (S10) and (S11) gives:

$$\Delta\chi^{\mathrm{ves}} = \frac{SP \cdot A \cdot t \cdot \rho^{\mathrm{PS}}}{M^{\mathrm{PS}}} \cdot \Delta\chi^{\mathrm{PS}} \qquad \text{(S12)}$$

With *SP* given by:

$$SP = \langle \frac{3\cos^2(\theta_N) - 1}{2} \rangle \qquad \text{(S13)}$$

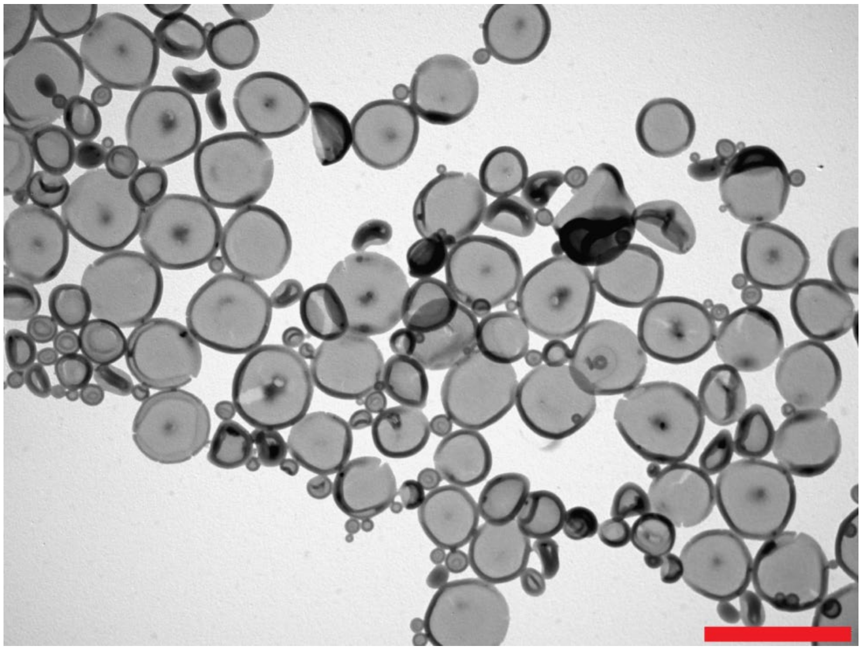

Supporting Figure 2: *TEM image of the PEG_{44}-b-PS_{178} discs. The whole sample consists of discs although there is some spread in the size of the discs. Scale bar is 1 µm.*

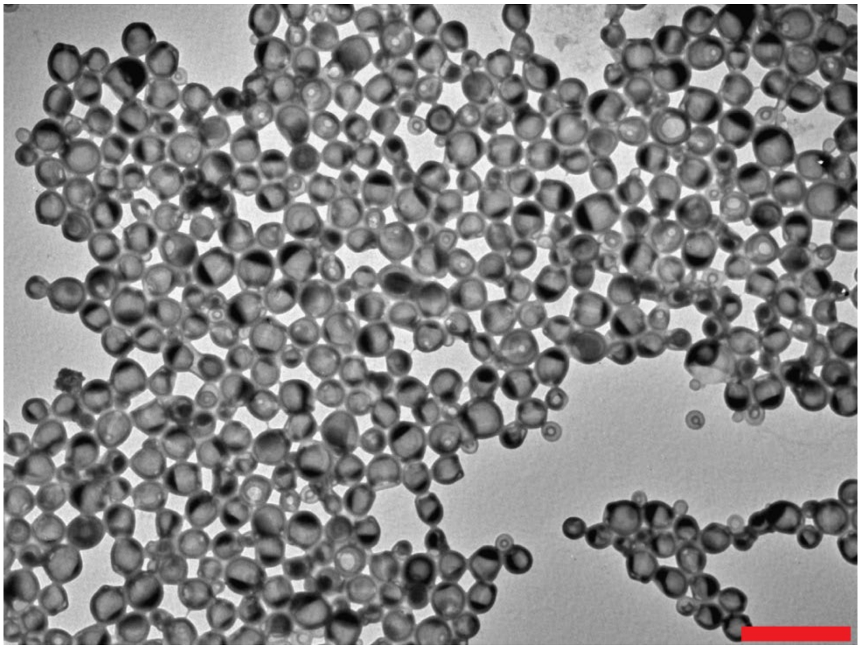

Supporting Figure 3: *TEM image of the PEG_{44}-b-PS_{178} stomatocytes. The whole sample consists of open stomatocytes although there is some small spread in the size and shape. Scale bar is 1 µm.*

Supporting Table 1: *Accuracy of the $\Delta\chi^{PS}$ of the magnetic birefringence curves for PEG_{44}-b-PS_{178} and PEG_{44}-b-PS_{195}.*

	R^2 for PEG_{44}-*b*-PS_{178}	R^2 for PEG_{44}-*b*-PS_{195}
Ellipsoids	n/a	0.9993
Tubes	n/a	0.9972
Discs	0.9997	0.9995
Stomatocytes	0.9991	n/a

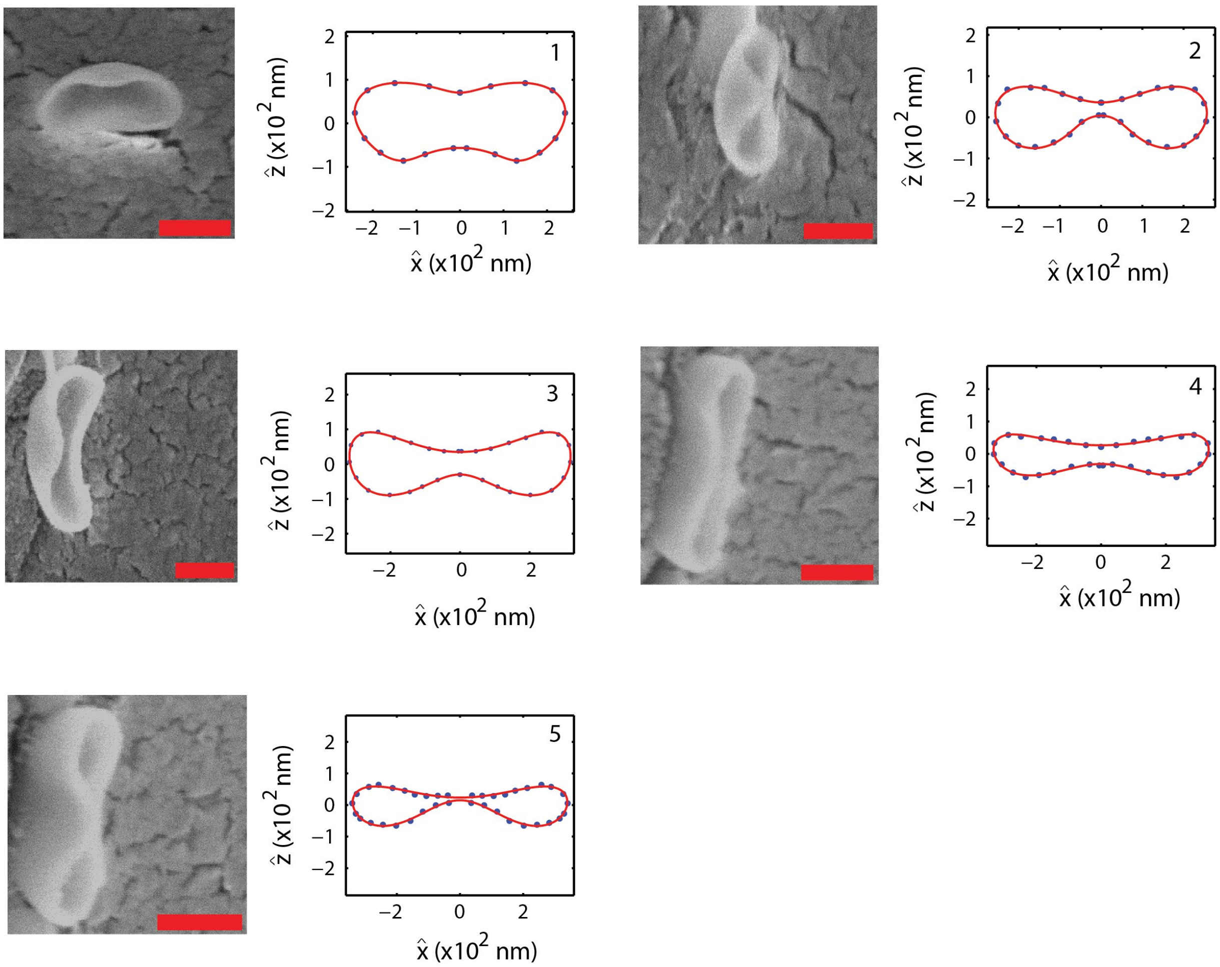

Supporting Figure 4: *Cryo-SEM images showing PEG_{44}-b-PS_{178} disc cross sections. Every cross section has been fitted using the parameterization stated in ref 1. The fitting parameters and the corresponding surface parameter (S_v) calculated are given in Supporting Table 2.*

#	a_1 ($\cdot 10^{-7}$ m)	a_2 ($\cdot 10^{-7}$ m)	a_3 ($\cdot 10^{-7}$ m)	a_4 ($\cdot 10^{-7}$ m)	a_5 ($\cdot 10^{-7}$ m)	b_1 ($\cdot 10^{-7}$ m)	b_2 ($\cdot 10^{-7}$ m)	b_3 ($\cdot 10^{-7}$ m)	b_4 ($\cdot 10^{-7}$ m)	b_5 ($\cdot 10^{-7}$ m)	$S_v \cdot A$ ($\cdot 10^{-14}$ m^2)
1	2.1385	0.1676	-0.1728	-0.1591	0.0000	0.9436	0.1059	-0.3093	-0.0370	0.0000	21.3
2	2.1213	0.5918	-0.1452	-0.1222	0.0000	0.4845	0.3580	-0.3229	-0.1589	0.0000	25.5
3	3.0176	0.3561	-0.1184	0.0298	0.0000	0.7837	0.0497	-0.4598	-0.0241	0.0000	40.0
4	3.2319	0.1119	-0.0888	0.0470	0.0000	0.5795	-0.0724	-0.2907	0.0491	0.0000	53.7
5	3.1649	0.5080	-0.0941	-0.0228	0.0000	0.4011	0.2411	-0.3582	-0.0488	0.0000	54.9

Supporting Table 2: Fitting parameters and surface parameter (S_v) for discs, obtained from the fittings as shown in Supporting Figure 4.

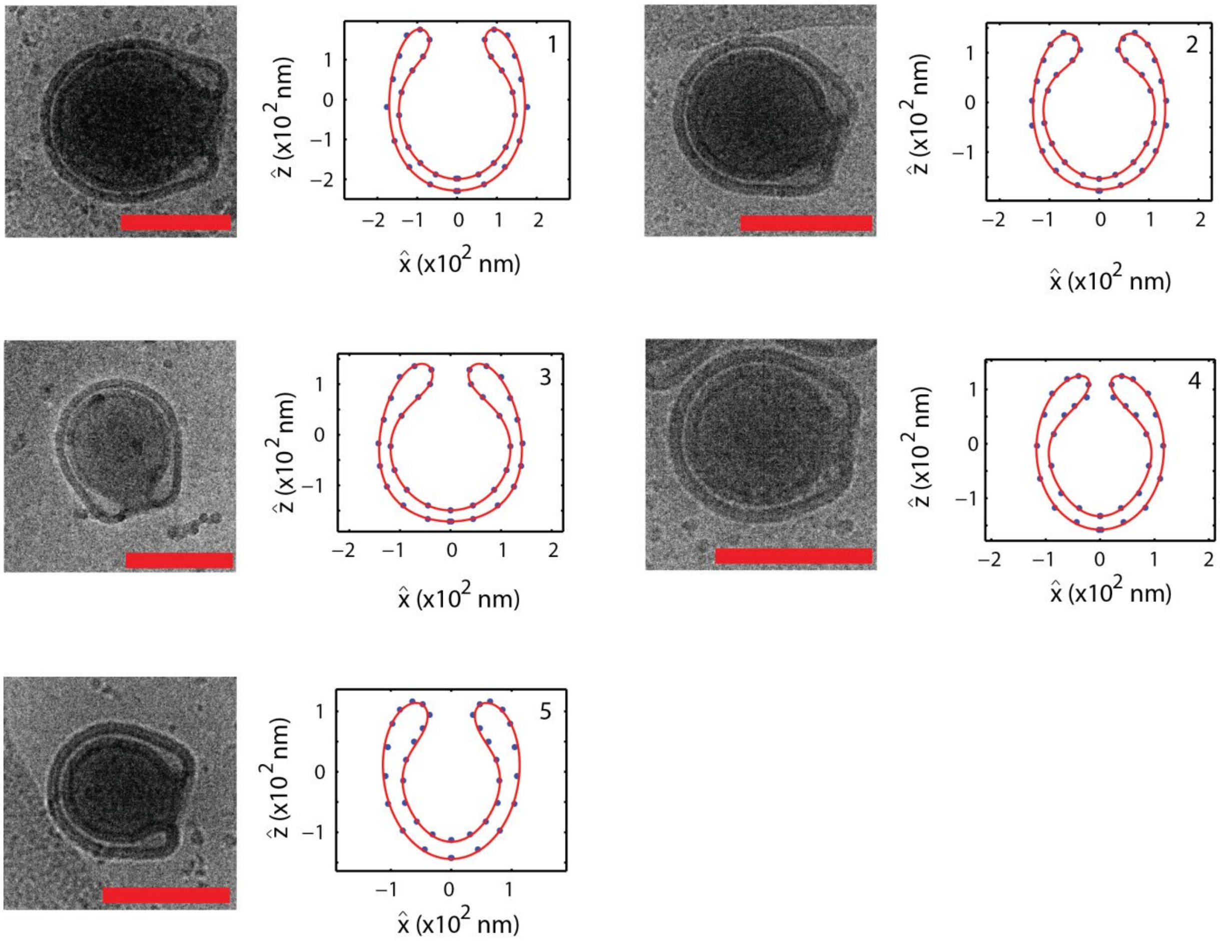

Supporting Figure 5: *Cryo-TEM images showing the cross sections of the PEG_{44}-b-PS_{178} stomatocytes. Every cross section has been fitted using the parameterization stated in ref 1. The fitting parameters and the corresponding surface parameter (S_v) calculated are given in Supporting Table 3.*

#	a_1 ($\cdot 10^{-7}$ m)	a_2 ($\cdot 10^{-7}$ m)	a_3 ($\cdot 10^{-7}$ m)	a_4 ($\cdot 10^{-7}$ m)	a_5 ($\cdot 10^{-7}$ m)	b_1 ($\cdot 10^{-7}$ m)	b_2 ($\cdot 10^{-7}$ m)	b_3 ($\cdot 10^{-7}$ m)	b_4 ($\cdot 10^{-7}$ m)	b_5 ($\cdot 10^{-7}$ m)	$S_v \cdot A$ ($\cdot 10^{-14}$ m^2)
1	1.4515	-0.1631	0.7525	0.0776	0.0478	0.0997	-1.8945	0.1776	-0.2429	-0.1324	-12.0
2	1.0529	-0.1408	0.6231	0.0700	0.0078	0.1055	-1.4522	0.1443	-0.1906	-0.1388	-5.68
3	1.0675	-0.1715	0.6920	0.0914	0.0345	0.1371	-1.4682	0.1001	-0.1342	-0.1271	-4.12
4	0.8132	-0.0521	0.5862	-0.1376	-0.0067	-0.1873	-1.2300	0.3174	-0.2222	-0.0065	-3.71
5	0.8653	-0.1414	0.4641	-0.0751	-0.0099	-0.1482	-1.1066	0.3026	-0.1917	-0.0122	-4.25

Supporting Table 3: Fitting parameters and surface parameter (S_v) for the stomatocytes, obtained from the fittings as shown in Supporting Figure 5.

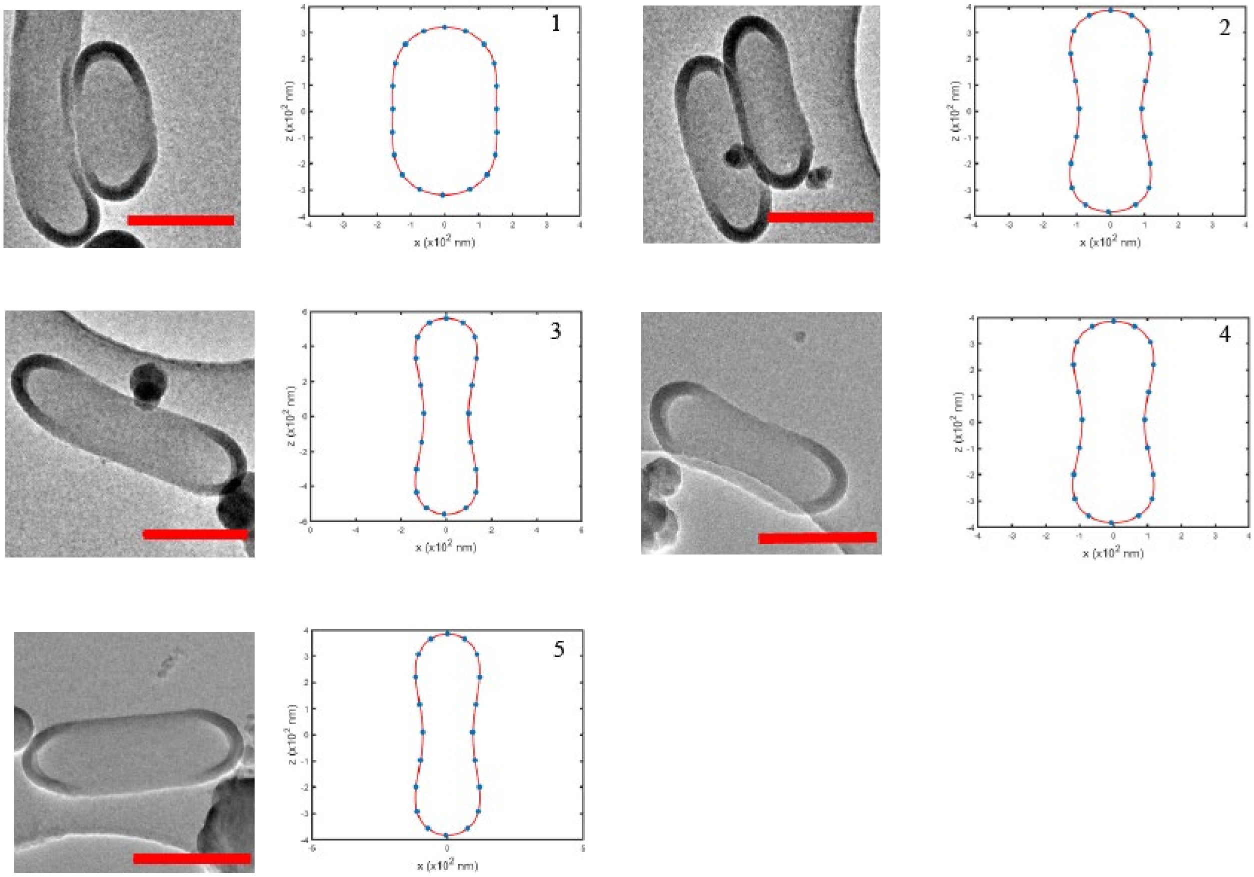

Supporting Figure 6: *Cryo-SEM images showing PEG$_{44}$-b-PS$_{195}$ tube cross sections. Every cross section has been fitted using the parameterization stated in ref 1. The fitting parameters and the corresponding surface parameter (S_v) calculated are given in Supporting Table 4.*

#	a_1 ($\cdot 10^{-7}$ m)	a_2 ($\cdot 10^{-7}$ m)	a_3 ($\cdot 10^{-7}$ m)	a_4 ($\cdot 10^{-7}$ m)	a_5 ($\cdot 10^{-7}$ m)	b_1 ($\cdot 10^{-7}$ m)	b_2 ($\cdot 10^{-7}$ m)	b_3 ($\cdot 10^{-7}$ m)	b_4 ($\cdot 10^{-7}$ m)	b_5 ($\cdot 10^{-7}$ m)	$S_v \cdot A$ ($\cdot 10^{-14}$ m^2)
1	1.7328	-0.0040	0.1906	0.0011	-0.0102	3.1115	0.0071	0.0954	0.0017	-0.0027	-16.1
2	1.2959	-0.0018	0.3643	0.0006	-0.0115	3.7290	0.0074	0.1036	0.0016	-0.0030	-16.7
3	1.5043	-0.0029	0.4316	0.0011	-0.0171	3.9473	0.0070	0.1778	0.0037	-0.0023	-19.8
4	1.5856	-0.0030	0.4549	0.0011	-0.0180	4.1607	0.0074	0.1874	0.0039	-0.0024	-22.1
5	1.5148	0.0030	0.4365	0.0053	-0.0391	4.7487	0.0324	0.0747	-0.0002	-0.0001	-25.5

Supporting Table 4: Fitting parameters and surface parameter (S_v) for tubess, obtained from the fittings as shown in Supporting Figure 6.

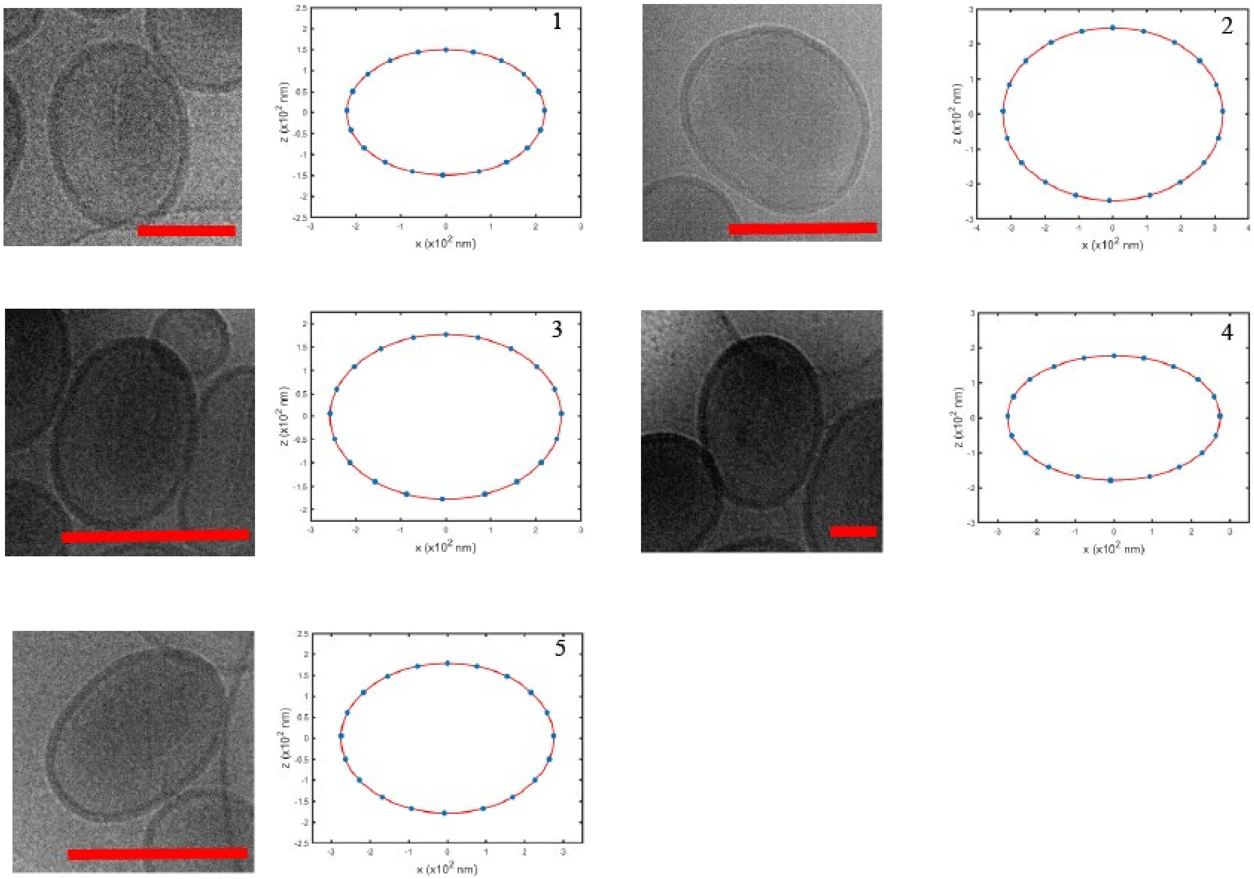

Supporting Figure 7: *Cryo-SEM images showing PEG_{44}-b-PS_{195} ellipsoid cross sections. Every cross section has been fitted using the parameterization stated in ref 1. The fitting parameters and the corresponding surface parameter (S_v) calculated are given in Supporting Table 5.*

#	a_1 ($\cdot 10^{-7}$ m)	a_2 ($\cdot 10^{-7}$ m)	a_3 ($\cdot 10^{-7}$ m)	a_4 ($\cdot 10^{-7}$ m)	a_5 ($\cdot 10^{-7}$ m)	b_1 ($\cdot 10^{-7}$ m)	b_2 ($\cdot 10^{-7}$ m)	b_3 ($\cdot 10^{-7}$ m)	b_4 ($\cdot 10^{-7}$ m)	b_5 ($\cdot 10^{-7}$ m)	$S_v \cdot A$ ($\cdot 10^{-14}$ m^2)
1	2.1901	0.0000	0.0001	0.0000	0.0001	1.4938	-0.0002	0.0003	-0.0016	0.0005	9.19
2	3.2375	0.0000	0.0001	0.0000	0.0000	2.4685	0.0001	-0.0002	-0.0003	0.0002	11.7
3	2.5632	0.0000	0.0001	0.0000	0.0000	1.7720	0.0001	-0.0002	-0.0001	0.0002	12.0
4	2.3376	0.0000	0.0001	0.0000	0.0000	1.5719	0.0001	-0.0002	0.0000	0.0002	11.9
5	2.7461	0.0000	0.0001	0.0000	0.0000	1.7821	0.0001	-0.0002	0.0000	0.0002	15.2

Supporting Table 5: Fitting parameters and surface parameter (S_v) for ellipsoids, obtained from the fittings as shown in Supporting Figure 7.

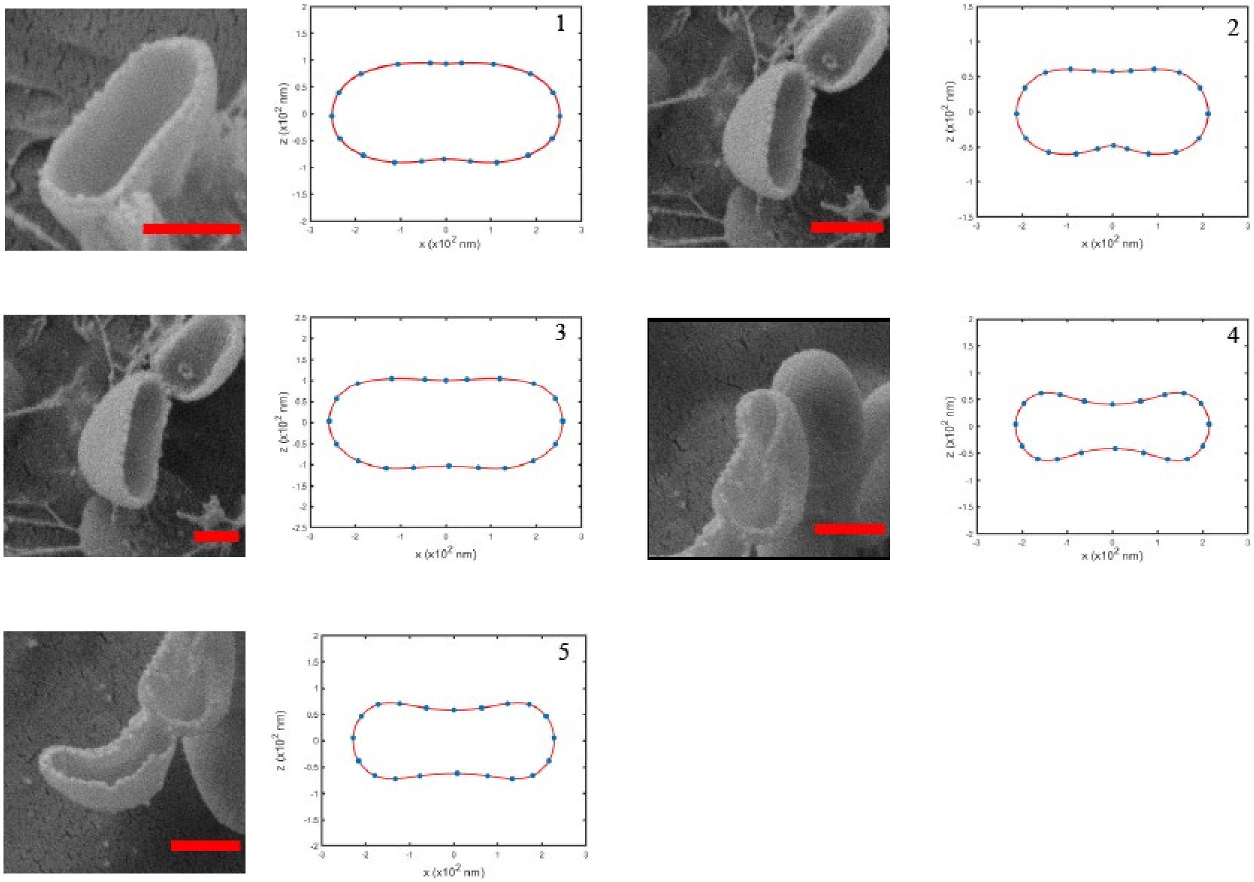

Supporting Figure 8: *Cryo-SEM images showing PEG$_{44}$-b-PS$_{195}$ disc cross sections. Every cross section has been fitted using the parameterization stated in ref 1. The fitting parameters and the corresponding surface parameter (S_v) calculated are given in Supporting Table 6.*

#	a_1 ($\cdot 10^{-7}$ m)	a_2 ($\cdot 10^{-7}$ m)	a_3 ($\cdot 10^{-7}$ m)	a_4 ($\cdot 10^{-7}$ m)	a_5 ($\cdot 10^{-7}$ m)	b_1 ($\cdot 10^{-7}$ m)	b_2 ($\cdot 10^{-7}$ m)	b_3 ($\cdot 10^{-7}$ m)	b_4 ($\cdot 10^{-7}$ m)	b_5 ($\cdot 10^{-7}$ m)	$S_v \cdot A$ ($\cdot 10^{-14}$ m^2)
1	2.3109	0.0505	-0.2661	-0.0458	-0.0560	1.0458	0.0649	-0.1458	-0.0010	-0.0164	26.2
2	1.8652	0.1104	-0.2510	0.0176	-0.0021	0.6845	0.0527	-0.1697	-0.0006	0.0084	24.9
3	2.4312	0.0218	-0.1740	-0.0498	-0.0239	1.2269	0.0040	-0.2053	0.0010	-0.0066	22.7
4	2.0632	0.0160	-0.0342	0.0111	0.0404	0.6508	0.0027	-0.2411	0.0004	0.0020	22.2
5	2.2170	-0.0004	-0.0275	-0.0023	0.0345	0.8103	-0.0109	-0.2109	-0.0022	0.0043	23.3

Supporting Table 6: Fitting parameters and surface parameter (S_v) for discs, obtained from the fittings as shown in Supporting Figure 8.

Supporting Information 2: Calculation of $\Delta\chi^{PS}$ as function of polymer extension

In a polymersome membrane the block-copolymers are not fully stretched since this configuration is statistically improbable. Rather, the block-copolymer will be coiled to some unknown extent. Therefore, one should investigate how the magnetic anisotropy depends on the degree of coiling. For this purpose, a Matlab script was written that calculates the magnetic anisotropy for PS as function of the degree of coiling. As an input parameter, the fraction of maximal extension is given, which determines the projection of the backbone C-C bonds on the *x*-axis. Then, all possible directions in the *y* and *z* directions are calculated, given the angles for certain bonds. From these possibilities, one is randomly chosen. This has no effect on the outcome of $\Delta\chi^P$, since the contributions in the *y* and *z* direction are averaged anyway. Again, the phenyl is allowed to rotate around the C-C bond connecting it to the backbone.

The result of the calculation is given in Supporting Figure 9a. It shows how the average magnetic anisotropy per repeating unit, $\Delta\chi^P$ depends on the degree of polymer extension. The contributions from the individual components are also given. The plot clearly shows that the magnetic anisotropy is dominated by the contribution of the phenyl group. This is mainly because the phenyl group has a much larger magnetic anisotropy than a C-C group. Furthermore, one can see that the contribution of the C-C bond connecting the phenyl to the backbone is of opposite of sign compared to the contributions of the backbone C-C's.[2,3] This makes the contribution of all C-C bonds together even smaller.

If the polymer gets more extended, $\Delta\chi^P$ will become more negative. Coiling will lead to a conformation in which the polymer occupies less space in the *x*-direction but more space in the *yz*-plane, as can be seen in Supporting Figure 6b and 6c. The effect is that $\Delta\chi^P$ becomes smaller upon coiling up to a point where it is actually zero. Upon further reduction of the projection of

the x-axis the sign of $\Delta\chi^P$ actually flips, meaning that the polymer becomes more extended in the yz-plane. In principle, this means that the polymer is stretched again, but this time in the yz-plane rather than in the x-direction.

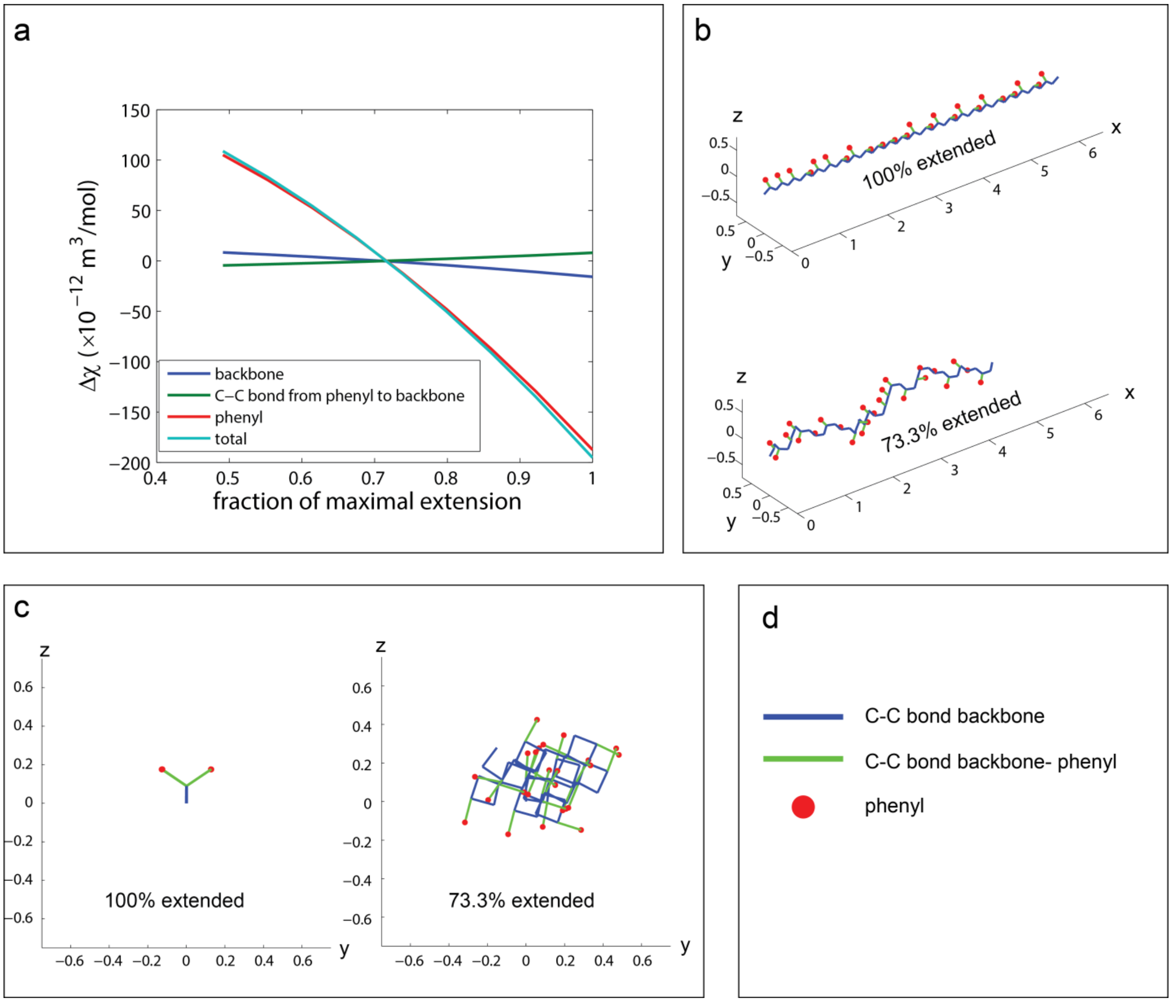

Supporting Figure 9: *Results of the calculation of the magnetic anisotropy of a single polystyrene repeating unit as function of the extension of the polystyrene polymer. (a) shows the contribution of the backbone, the C-C bond connecting the phenyl to the backbone and the phenyl group itself, which is allowed to rotate freely around the C-C bond connecting it to the backbone. The sum of all contributions is shown as well. The extension of a polystyrene polymer is illustrated in (b) and (c) for a PS chain consisting of 24 units. The more extended the polymer is, the more the bonds will project in similar directions, leading to a more negative magnetic anisotropy as is calculated in (a). (d) Legend.*

References

1 Rikken, R. S. *et al.* Shaping polymersomes into predictable morphologies via out-of-equilibrium self-assembly. **7**, 1-7 (2016).

2 Hellwege, K. H., Gupta, R. R. & Hellwege, A. M. *Diamagnetic Susceptibility / Diamagnetische Suszeptibilität*. (Springer Berlin Heidelberg, 1986).

3 Maret, G., Dransfeld, K. J. S., Fields, U. M. & Applications, T. Biomolecules and polymers in high steady magnetic fields. 143-204 (2005).
